# Identification of Key Genes of Prognostic Value in Clear Cell Renal Cell Carcinoma Microenvironment and a Risk Score Prognostic Model

**DOI:** 10.1155/2020/8852388

**Published:** 2020-09-03

**Authors:** Enfa Zhao, Xiaofang Bai

**Affiliations:** ^1^Department of Structural Heart Disease, The First Affiliated Hospital of Xi'an Jiaotong University, Xi'an 710061, China; ^2^Department of Ultrasound Medicine, The First Affiliated Hospital of Xi'an Jiaotong University, Xi'an 710061, China

## Abstract

**Objective:**

We aimed at identifying the key genes of prognostic value in clear cell renal cell carcinoma (ccRCC) microenvironment and construct a risk score prognostic model.

**Materials and Methods:**

Immune and stromal scores were calculated using the ESTIMATE algorithm. A total of 539 ccRCC cases were divided into high- and low-score groups. The differentially expressed genes in immune and stromal cells for the prognosis of ccRCC were screened. The relationship between survival outcome and gene expression was evaluated using univariate and multivariate Cox proportional hazard regression analyses. A risk score prognostic model was constructed based on the immune/stromal scores.

**Results:**

The median survival time of the low immune score group was longer than that of the high immune score group (*p* = 0.044). Ten tumor microenvironment-related genes were selected by screening, and a predictive model was established, based on which patients were divided into high- and low-risk groups with markedly different overall survival (*p* < 0.0001). Multivariate Cox analyses showed that the risk score prognostic model was independently associated with overall survival, with a hazard ratio of 1.0437 (confidence interval: 1.0237–1.0641, *p* < 0.0001).

**Conclusions:**

Low immune scores were associated with extended survival time compared to high immune scores. The novel risk predictive model based on tumor microenvironment-related genes may be an independent prognostic biomarker in ccRCC.

## 1. Introduction

Renal cancer, the most common lethal genitourinary cancer, accounts for up to 2–3% of all adult malignancies worldwide [1]. Clear cell renal cell carcinoma (ccRCC) is the most common pathological subtype, accounting for approximately 65–70% of renal neoplasms [2]. It is a relatively low-grade cancer, and patients are generally asymptomatic in the early stages. Symptoms are obvious when tumor volume is large and the cancer has reached the advanced stages [3]. With rapid advancements in clinical diagnosis as well as treatment strategies, the overall survival rate of ccRCC patients has increased. However, due to a delay in diagnosis, and local or distant metastasis, the overall survival of 30% of the newly diagnosed ccRCC patients, who present with metastasis at the time of diagnosis, is approximately 13 months [4, 5]. Currently, prognosis is based on the pathological stage and grade of cancer in patients with renal cancers [6]. Despite significant advances in early detection, surgery, and medical treatment, the prognosis of ccRCC remains unsatisfactory. Therefore, more effective biomarkers and therapeutic targets are urgently needed.

It is widely known that tumor tissues consist of tumor cells as well as tumor-related normal epithelial, immune, stromal cells, and vascular cells [7]. In ccRCC, cells in the tumor microenvironment (TME) promote growth and metastasis of tumor cells and suppress the immune system via several elaborate mechanisms [8]. Constituents of the TME include tumor cells, immune cells, and various types of stromal cells; their interactions have a significant effect on treatment response and disease prognosis. TME also significantly influences carcinogenesis, gene expression in the cancer tissues, and clinical prognosis [7, 9–11]. TME cells constitute important components of the cancer tissue. TME is the cellular milieu where the tumor is located. Tumors are usually formed by aggregates of various tumor cell types, and immune and stromal cells, which constitute the two main nontumor cell types in the TME [12, 13]. To quantify the cellular composition of the TME, an algorithm called ESTIMATE (Estimation of Stromal and Immune cells in Malignant Tumor tissues using Expression data) was used to determine the composition of immune and stromal cells in tumor samples [7]. ESTIMATE is a tool that predicts tumor purity and the level of infiltrating immune/stromal cells in tumor tissues using gene expression data. ESTIMATE scores are significantly related to tumor purity in various cancer samples. In this algorithm, immune and stromal cell levels were chosen to predict the infiltration of nontumor cells by analyzing their specific gene expression signatures. Stromal score indicates the presence of stroma in the tumor tissue, while the immune score indicates the infiltration of immune cells in tumor tissue; the estimated score indicates tumor purity. A positive correlation was reported between immune and stromal scores, and samples with high tumor purity showed low immune and stromal scores [7]. Previous studies have used the ESTIMATE algorithm to evaluate the prognostic value of immune and stromal cells in several cancers [14–16]. The TME is increasingly being considered to play a vital role in tumor growth. Therefore, understanding the components of stromal and immune microenvironments may contribute to improve prognosis and customized therapies. However, whether TME contributes to ccRCC survival has not been well studied. In this study, we aimed at identifying key TME-related genes of prognostic value in ccRCC and generate a risk score prognostic model.

## 2. Materials and Methods

Level 3 raw microarray mRNA expression data pertaining to 539 ccRCC samples were downloaded from the TCGA database (https://portal.gdc.cancer.gov/repository) on April 3, 2019. The clinicopathological data of 537 tumor patients, including sex, age, clinical stage, T-stage, lymph node involvement, survival outcome, and survival (duration in days) were also downloaded and reorganized for further analysis. ESTIMATE is known to predict the immune and stromal scores of cells by performing single sample Gene Set Enrichment Analysis (ssGSEA), which forms the basis of the ESTIMATE score [17, 18]. The scores for all TCGA tumor types are available online in the MD Anderson Cancer Center website (https://bioinformatics.mdanderson.org/estimate/). Stromal and immune scores of each ccRCC sample were determined by using the ESTIMATE algorithm using the estimate package provided on R-Forge for all tumor samples [7].

### 2.1. Identification of Differentially Expressed Genes (DEGs)

We categorized all patients into high and low-score groups according to their median immune/stromal scores. The limma package in R software with multiple testing corrections based on the Benjamini and Hochberg method was employed to screen the DEGs [19]. The criteria for screening of upregulated DEGs were defined as log_2_FC > 1 and adjusted *p* value < 0.05, while that for downregulated DEGs were defined as log_2_FC < −1 with adjusted *p* value < 0.05. For comparison based on immune and stromal scores, upregulated and downregulated genes were identified for both. Lastly, the common upregulated and downregulated DEGs in the stromal and immune score groups were identified using an online Venn diagram and defined as upregulated and downregulated DEGs.

### 2.2. Overall Survival Curve

Kaplan-Meier plots, tested using the log-rank method, were used to establish a potential relationship between prognostic values and gene expression levels of the identified DEGs between the high and low immune/stromal score groups in patients.

### 2.3. Construction of the Risk Score Prognostic Model in the Training Cohort

The genes tested using the Kaplan-Meier plots with a *p* value < 0.05 were considered for further analysis. A total of 530 patients with complete clinical information and mRNA expression data were enrolled in the study. We first randomly assigned 264 patients to the training cohort, while the entire TCGA cohort was used as the validation cohort. Next, a total of 43 genes associated with overall survival were subjected to further selection using univariate Cox proportional hazard regression analysis in the training cohort. Only genes with a *p* value < 0.05 were regarded as possible variables and used in multivariate Cox regression analysis to build the risk predictive model.

(1) $$\begin{array}{c} \text{Risk score}=­\left({\text{Expr}}_{\text{gene}-1}\times {\text{Coef}}_{\text{gene}-1}\right)+­\left({\text{Expr}}_{\text{gene}-2}\times {\text{Coef}}_{\text{gene}-2}\right)+­\left({\text{Expr}}_{\text{gene}-\text{n}}\times {\text{Coef}}_{\text{gene}-\text{n}}\right), \end{array}$$

where Expr is the expression level of gene and Coef is the regression coefficient derived from the multivariate Cox regression model.

Each patient was assigned a risk score according to the above risk predictive model. Patients were then further divided into high- and low-risk groups using the median value as the cutoff point. Time-dependent receiver operating characteristic (ROC) curve analysis was performed to determine the power of the predictive model.

### 2.4. Enrichment Analysis of the DEGs

To identify the potential gene ontology (GO) categories by their biological processes (BP), molecular functions (MF), cellular components (CC), and to determine the Kyoto Encyclopedia of Genes and Genomes (KEGG) signaling pathways associated with all the upregulated and downregulated DEGs, clusterProfiler and DOSE package in Bioconductor (https://bioconductor.org/) were used to perform GO and KEGG pathway analyses. Results of the GO and KEGG analyses were regarded as statistically significant at *p* < 0.05.

## 3. Results

### 3.1. Summary of Immune and Stromal Scores from ccRCC Patients

**Figure 1:**
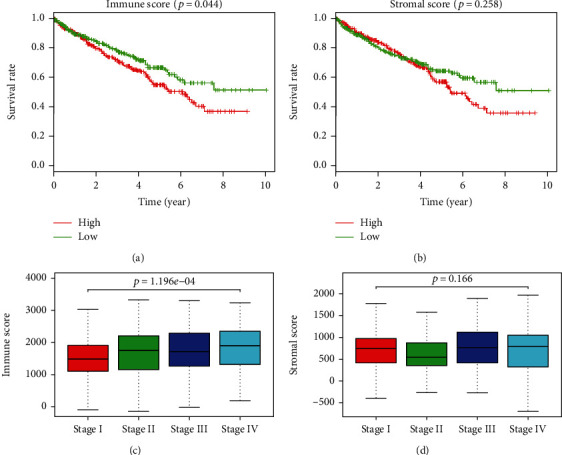
Immune and stromal scores, and their relationship with overall survival. (a) Patients were divided into two groups based on their immune scores: 267 cases presented high immune scores and 263 presented low immune scores; (b) Patients were divided into two groups based on their stromal scores: 264 cases presented high stromal scores and 266 presented low stromal scores. (c) Distribution of immune scores in patients in stages I, II, III, and IV of clear cell renal cell carcinoma. (d) Distribution of stromal scores in patients in stages I, II, III, and IV of clear cell renal cell carcinoma.

A total of 539 mRNA profiles and 537 clinical information data pertaining to ccRCC patients were downloaded from the TCGA database. Among patients, 336 (62.57%) patients were <65 years old and 201 (37.43%) were ≥65 years old. A total of 191 (35.57%) patients were females and 346 (64.43%) were males. The mean follow-up time was 3.11 years. During the mean follow-up time, 82 of the total 264 patients in the training cohort and 166 of 530 patients (TCGA entirely) in the validation cohort died. There was no statistical difference with respect to overall survival (*p* = 0.938) between cohorts. With respect to the clinical-pathological stage, 265 patients were in stage I, 57 in stage II, 123 in stage III, and 82 in stage IV; the staging of three patients was unknown (Table 1). Based on the ESTIMATE algorithm mentioned above, the immune scores ranged between -693.95 and 3328.21 (with a median of 1604.4), and the stromal scores ranged between -1433.77 and 1967.19 (with a median of 728.66). The 537 ccRCC patients in the study were classified into low and high immune/stromal score groups using their median immune/stromal scores as cutoff points. Kaplan-Meier survival curve of the immune scores revealed that the median survival time of the high-score group was associated with shorter survival time than that of the low-score group (*p* = 0.044), as evidenced by log-rank test (Figure 1(a)). Similarly, the Kaplan-Meier survival curve of stromal scores revealed that the high-score group was associated with shorter median survival time than that of the low-score group (although the difference was not statistically significant), as indicated by log-rank test (*p* = 0.258; Figure 1(b)). Furthermore, the average immune score of stage IV patients ranked highest among all stages, followed by stages III and II. Stage I patients received the lowest immune scores, as revealed by the Wilcoxon signed-rank test (Figure 1(c)). Although the results were statistically significant, there was a significant overlap in the median values as well as the range of the immune scores among the four groups of tumor stages. Moreover, these differences were only marginally significant, and therefore the results must be interpreted cautiously. However, this association was not found in the stromal scores (Figure 1(d)).

### 3.2. Gene Expression Profiles in the Stromal and Immune Score Groups of ccRCC Patients

**Figure 2:**
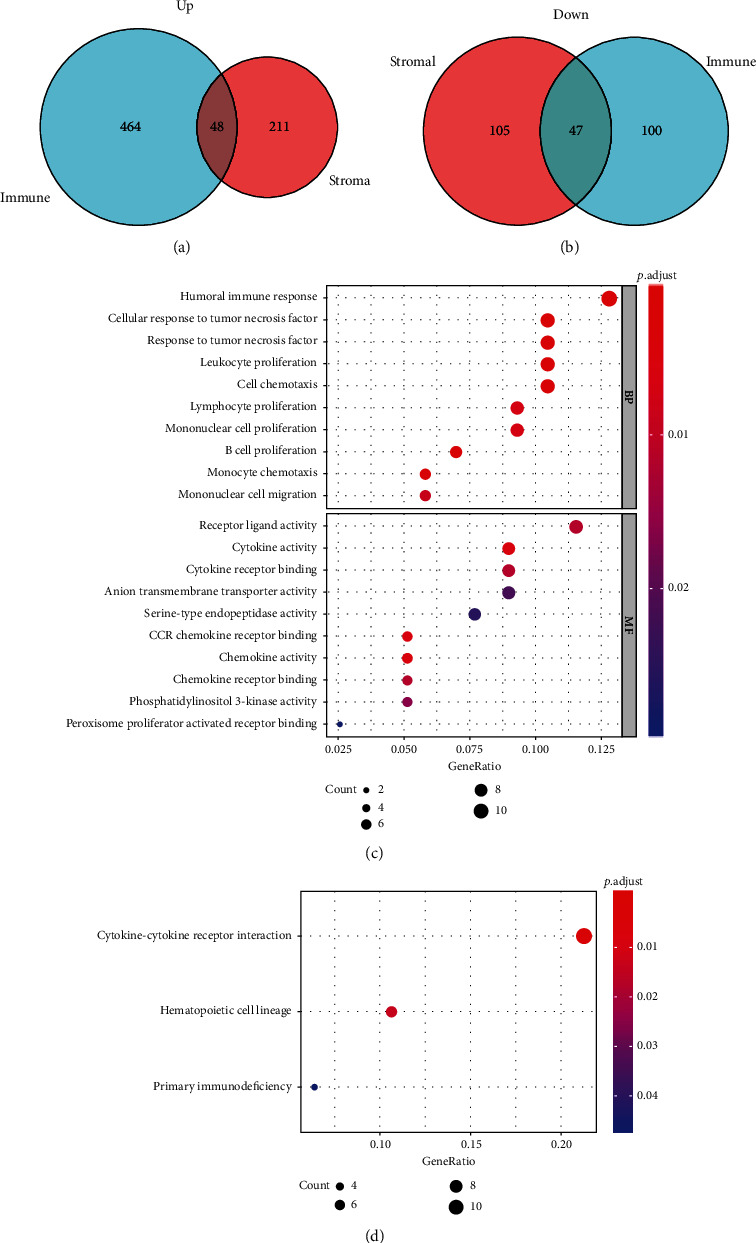
Gene expression profile with stromal and immune scores patients with clear cell renal cell carcinoma. (a) Venn diagrams indicating the number of upregulated DEGs in the stromal and immune score groups; (b) Venn diagrams revealing the number of downregulated DEGs in the stromal and immune score groups; (c) Top ten gene ontology terms of the 95 differentially expressed genes; (d) KEGG analysis of the 95 differentially expressed genes.

A total of 512 upregulated and 147 downregulated DEGs were identified in the immune score group, while 259 upregulated and 152 downregulated DEGs were identified in the stromal score group. Furthermore, Venn diagrams revealed that 48 upregulated genes and 47 downregulated genes were common between the two high-scores groups (Figures 2(a) and 2(b)). To investigate the potential biological functions of the 95 identified DEGs, KEGG pathway enrichment and GO functional analyses were performed. The top GO terms identified included humoral immune response, cell chemotaxis, chemokine activities, cytokine-cytokine receptor interaction, and primary immunodeficiency (Figures 2(d) and 2(d)).

### 3.3. Identification of the Effect of the Individual DEGs on Overall Survival

To explore the potential roles of the 95 identified DEGs in overall survival, Kaplan-Meier survival curves were used to establish the potential relationship between the prognostic roles and gene expression levels. Among the 95 DEGs, a total of 43 DEGs (Supplementary Table S1) were found to be significantly related to overall survival in the log-rank test.

### 3.4. Establishment of Risk Score Prognostic Model

**Figure 3:**
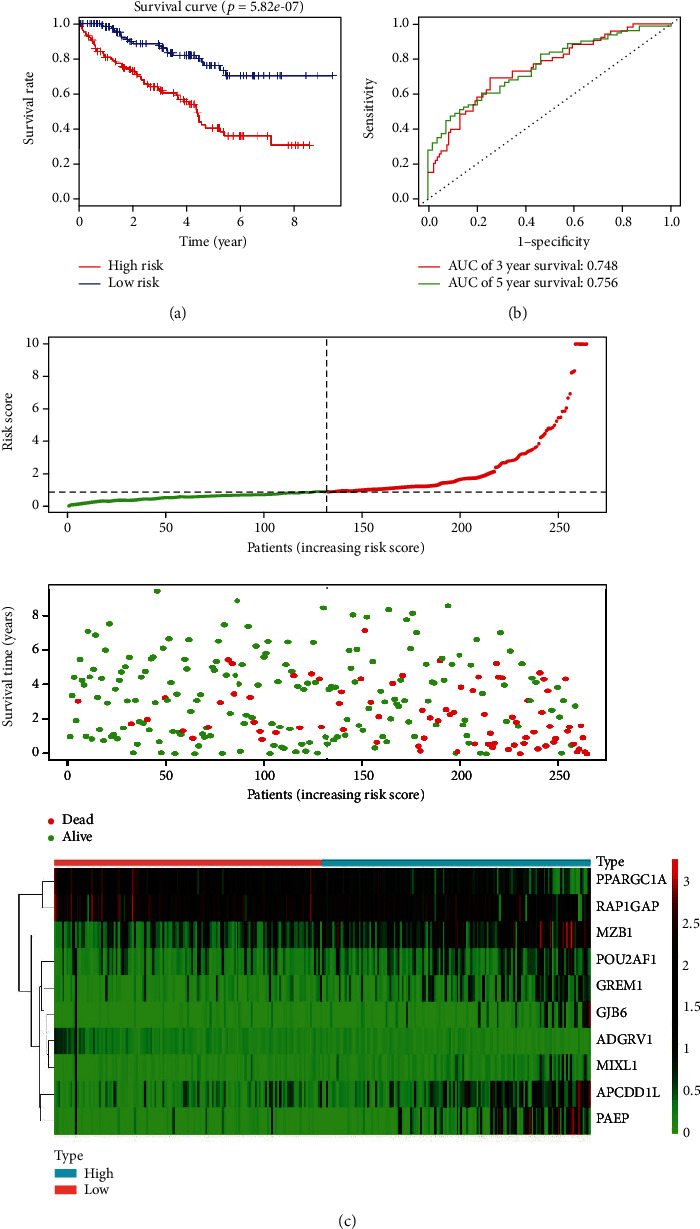
Prognostic value of the ten gene signature in the training cohort of patients with clear cell renal cell carcinoma patients. (a) Kaplan-Meier risk survival curve of high- and low-risk patients grouped according to median risk score; (b) Receiver operating characteristic curve analysis of 3-year and 5-year overall survival of the ten-gene signature; (c) Distribution of survival status, risk score, and gene expression level in patients with clear cell renal cell carcinoma. Patient subgroups with high and low-risk scores were classified using the optimal cutoff value. Dotted line indicates the cutoff point of the median risk score used to categorize patients into low- and high-risk groups.

Univariate Cox regression analysis demonstrated that 33 DEGs were significantly associated with overall survival (*p* < 0.05) among the 43 DEGs. To construct a predictive model with optimal efficacy and sufficient information, the 33 candidate genes, including APCDD1L, GJB6, CASP5, SLN, HSD11B1, PPARGC1A, ZPLD1, SLC22A12, SLC22A6, HMGCS2, CPA4, ADGRV1, GPAT3, PAEP, MZB1, RORB, IGLL5, OGDHL, AQP9, LDHD, FDCSP, HSD11B2, TNFSF13B, FREM1, FCRL5, POU2AF1, MUC20, VSIG4, RAP1GAP, MIXL1, GREM1, PAH, and SLC22A8, were fitted into a multivariable Cox proportional hazards regression analysis in the training cohort. Ten survival-related genes displayed a significant prognostic value for ccRCC. We then constructed a prognostic signature based on the expression levels of these ten genes and their coefficients derived from the multivariable Cox model. The risk score prognostic model was computed as follows: Risk score = (−3.1648 ∗ expression value of ADGRV1) + (0.2204 ∗ expression value of APCDD1L) + (0.3514 × ∗ expression value of GREM1) + (0.5583 ∗ expression value of GJB6) + (0.4944 ∗ expression value of MZB1) + (−1.1655 ∗ expression value of POU2AF1) + (0.2392 ∗ expression value of RAP1GAP) + (0.1515 ∗ expression value of PAEP) + (1.2305 ∗ expression value of MIXL1) + (−0.3431 ∗ expression value of PPARGC1A). This equation clearly demonstrated that APCDD1L, GREM1, GJB6, MZB1, RAP1GAP, PAEP, and MIXL1 constituted risk factors for ccRCC prognosis (coefficient > 0), while ADGRV1, POU2AF1, and PPARGC1A constituted protective factors (coefficient < 0). The value of their respective coefficients indicated the extent of their impact on survival prediction. MIXL1 presented the highest risk, while ADGRV1 had the most protective effect. Patients with a ten-gene signature risk score higher than the median risk score were classified as high-risk, while those with a risk score lower than the median risk score were classified as low-risk. The Kaplan-Meier overall survival curve of the high-risk group was significantly lower than that of the low-risk group (log-rank *p* < 0.0001; Figure 3(a)). Univariate analysis revealed that the ten-gene signature correlated significantly with poor overall survival (hazard ratio [HR]: 1.0509; 95% confidence interval [CI]: 1.0283–1.0740; *p* < 0.0001). Multivariate Cox analyses showed that the ten-gene signature remained independently associated with overall survival, with an HR of 1.0437 (CI: 1.0237–1.0641, *p* < 0.0001), along with age, grade, and stage (Table 2), revealing that the risk score prognostic model may be an independent predictor of overall survival. Furthermore, to estimate the prognostic risk score model for 3 and 5 years, the area under the receiver operating characteristic (ROC) curve (AUC) was calculated. As shown in Figure 3(b), the AUC of the ten-gene model for survival prediction was 0.748 at 3 years of overall survival and 0.756 at 5 years of overall survival. The distribution of risk score, survival status, and ten-gene signature expression of individual patients was further analyzed. Patient subgroups with high and low-risk scores were classified using the optimal cut-off. An increased risk score was associated with higher patient death rate (Figure 3(c)).

### 3.5. Validation of the Risk Score Prognostic Model in TCGA Cohort

**Figure 4:**
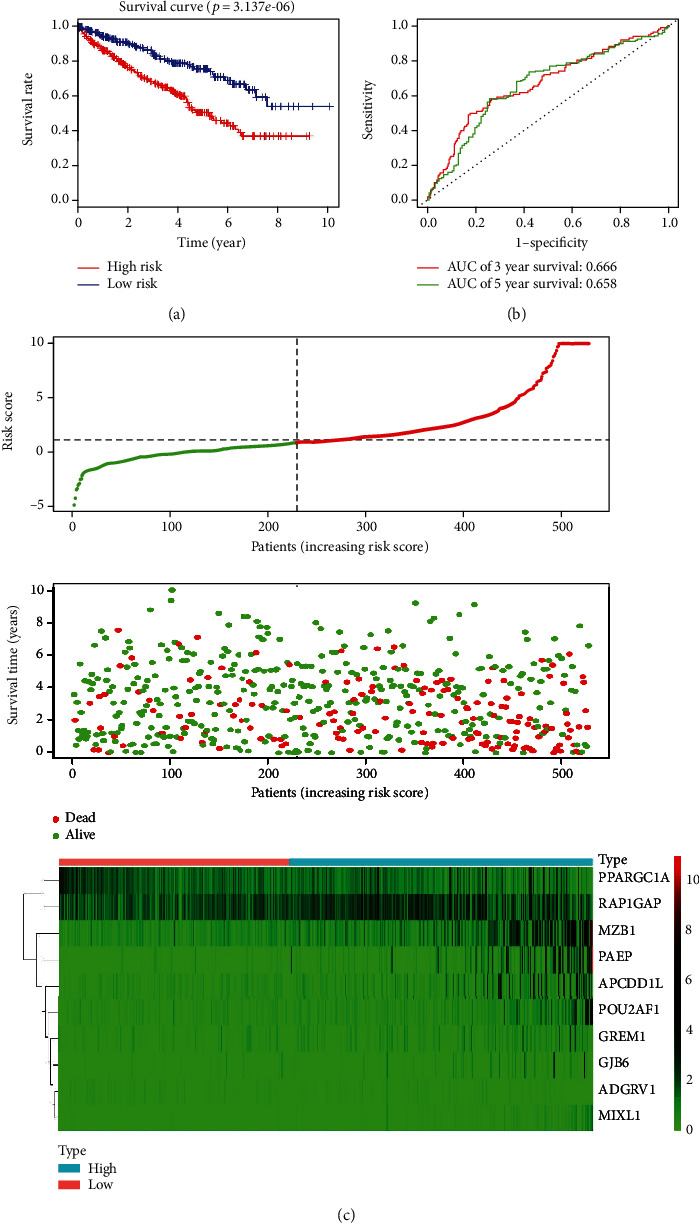
Prognostic value of the risk score prognostic model based on ten immune-related genes in the entire TCGA cohort of patients with clear cell renal cell carcinoma. (a) Kaplan-Meier risk survival curve of high- and low-risk patients grouped according to median risk score; (b) Receiver operating characteristic curve analysis of 3-year and 5-year overall survival of the ten-gene signature; (c) Distribution of survival status, risk score, and gene expression level in patients with clear cell renal cell carcinoma. Patient subgroups with high- and low-risk scores were classified by the optimal cutoff value. The dotted line indicates the cutoff point of the median risk score used to categorize patients into low- and high-risk groups.

To test the robustness of the prediction power of the ten-gene signature, we extended the testing to the TCGA validation cohort entirely. The risk score of each patient in the validation cohort was calculated based on the expression value of the ten-gene signature and was divided into the high- or low-risk groups according to the cutoff point of the median risk score (0.8768) derived from the training cohort. Using the same risk score model and cutoff point, 230 patients were classified into the low-risk group and 300 patients were classified into the high-risk group. Kaplan-Meier survival curves of the two groups based on the ten-gene signature were notably different in the validation cohort. Patients in the high-risk group had obviously shorter overall than those in the low-risk group (*p* < 0.0001, Figure 4(a)). By calculating the AUC of the ROC curve of the risk score, we could predict the 3-year and 5-year survival rates of patients with ccRCC (0.666 for 3 years and 0.658 for 5 years; Figure 4(b)). Distribution of the ten-gene signature risk scores, expression pattern of prognostic signature, and survival status are shown in Figure 4(c) and are consistent with findings in the training cohort. Combining the results of the training and total cohorts, the ten-gene signature was demonstrated to be an effective independent prognostic biomarker in patients with ccRCC.

## 4. Discussion

Increasing evidence has revealed a close interplay between the tumor and nontumor components that contribute to increased angiogenesis, invasion, progression, and metastasis in several tumors, such as pancreatic, breast, and lung cancers, and glioblastoma [20–23]. Stromal and immune microenvironments suppress the tumor and prevent the metastasis as well as permeability of several drugs into the tumor [24]. The tumor microenvironment can stimulate angiogenesis and tumor cell survival, resulting in poor prognosis [25, 26]. Our study results were consistent with previous findings and revealed an association between immune and stromal microenvironments and tumor progression. These mechanisms have not been completely explored and warrant future investigations.

In this study, we aimed at determining the role of TME-related genes in the overall survival in ccRCC based on the TCGA database. We classified all patients with ccRCC into low and high immune score groups based on their median immune scores. The Kaplan-Meier survival analysis revealed that the median survival time of the high immune score group was shorter than that of the low-score group, indicating that TME-related immune cells can be used to categorized patients into high and low-score groups with notably different overall survival. This finding was inconsistent with the previous view that tumor immunity suppresses tumor cells. The TME is a mixture of fluids, stromal cells, immune cells, extracellular matrix molecules, and numerous cytokines and chemokines, coupled with their significant interactions. However, cells and molecules in this environment are in a dynamic state and contribute to tumor immune evasion, tumor growth, and metastasis, thereby showing the evolutionary nature of tumors [8]. TME-induced metabolic stress on infiltrating immune cells can result in local immunosuppression and limited reinvigoration of antitumor immunity and lead to impaired antitumor immune responses [27].

TME is a factor that drives the carcinogenesis of various cancers. In pancreatic adenocarcinoma (PAAD), high- and low-immune score groups were identified using the ESTIMATE score. Kaplan-Meier curves revealed significantly worse survival of patients with high-risk scores in both the training and validation groups [15]. Higher scores were associated with worse survival outcomes for immune scores (*p* = 0.0167), stromal scores (*p* = 0.0035), and ESTIMATE scores (*p* = 0.0190) in lower-grade glioma [14]. The potential prognostic value of immune and stromal scores in stomach adenocarcinoma has been confirmed. High stromal and immune scores were reported to be associated with poor overall survival (*p* = 0.0032 and *p* = 0.05, respectively). The estimated score was also related to overall survival (*p* = 0.0359) [16]. In ccRCC, a higher immune score was associated with shorter overall survival (*p* = 0.04), and a close association was reported between immune scores, clinical characteristics, as well as prognosis in ccRCC [28]. These data indicate that TME plays an important role in tumorigenesis and prognosis.

KEGG pathway enrichment and GO functional analyses of these genes revealed that they were mainly involved in humoral immune response, cell chemotaxis, chemokine activities, cytokine-cytokine receptor interaction, and primary immunodeficiency. We also performed a Kaplan-Meier analysis of these genes and identified 33 immune-related genes that were associated with different outcomes in patients with ccRCC. Of these, several genes including CASP5, RAP1GAP, and GREM1 have been reported to be involved in the pathogenesis of renal cancer or significant in predicting overall survival, suggesting that the present analysis using the TCGA database has potential prognostic value [29–32]. Further, using univariate and multivariate Cox proportional hazard regression analysis, a ten immune-related gene risk score prognostic model was established. The Kaplan-Meier overall survival of the risk score prognostic model in the high-risk group was significantly shorter than that in the low-risk group, revealing that the risk score prognostic model based on immune-related genes may be an independent predictor of overall survival. The prognostic power of the risk score model was evaluated by calculating the AUC of the ROC curve. Higher AUC demonstrates good model performance. The AUC for the ten-gene model for survival prediction was 0.703 at 3 years of overall survival and 0.715 at 5 years of overall survival. The performance of the prognostic model was further confirmed in the entire cohort, revealing its superior performance in predicting ccRCC patient survival.

Several limitations of the study need to be noted. First, the TME signature was analyzed and validated only in the TCGA data set, and no other ccRCC-related expression profiles including prognostic clinical information were available for further validation. Second, no experimental data were obtained. Further experimental studies are needed to improve our understanding of the functional role of immune-related genes in ccRCC.

## 5. Conclusion

Based on a comprehensive analysis of TME-related genes, our results indicate that the assessment of the immune and stromal status using the TME signature is an optimal predictor of survival in ccRCC patients. The novel risk predictive model proposed in this study constitutes an effective independent prognostic biomarker in patients with ccRCC. These findings have promising clinical implications for improving the outcome prediction for ccRCC patients contingent upon future validation.

## Figures and Tables

**Table 1:** Clinical characteristics of patients in the training and validation cohorts.

Variables	Train cohort (*N* = 264)	Validation cohort (*N* = 530)	*p* value
Age (years)	60.19 ± 11.83	60.56 ± 12.14	0.6834
Gender (female vs. male)			0.6476
Female	97	186	
Male	167	344	
Grade			0.9749
I	9	14	
II	110	227	
III	105	206	
IV	36	75	
Unknown	4	8	
Stage			0.9768
I	132	265	
II	25	57	
III	62	123	
IV	43	82	
Unknown	2	3	
T classification			0.9619
T1	136	271	
T2	31	69	
T3	91	179	
T4	6	11	
M classification			0.8932
M0	206	420	
M1	40	78	
Unknown	18	32	
N classification			0.6492
N0	111	239	
N1	10	16	
Unknown	143	275	
Survival status			0.9383
Dead	82	166	
Alive	182	364	

**Table 2:** Univariate and multivariate analysis of the correlation of the ten-gene signature with overall survival in clear cell renal cell carcinoma patients.

		Univariate analysis			Multivariate analysis	
Parameter	HR	95% CI	*p* value	HR	95% CI	*p* value
Age	1.0328	1.0191-1.0468	**2.29*E*-06**	1.0344	1.0190-1.0501	**1.02*E*-05**
Gender	0.9311	0.6754-1.2836	0.6629	0.9577	0.6893-1.3305	0.796641
Grade	2.2931	1.8541-2.8360	**1.94*E*-14**	1.4751	1.1610-1.8743	0.001465
Stage	1.8888	1.64882.1637	**4.67*E*-20**	1.7651	1.1269-2.7646	0.013065
T	1.9414	1.6393-2.2991	**1.50*E*-14**	0.8648	0.5744-1.3022	0.486758
M	4.2835	3.10575.9080	**7.45*E*-19**	1.2009	0.6127-2.3538	0.593805
Ten-gene signature	1.0509	1.0283-1.0740	**7.71*E*-06**	1.0437	1.0237-1.0641	**1.48*E*-05**

Bold values indicate *p* < 0.05; HR: hazard ratio; CI: confidence interval.

## Data Availability

The raw data of this study are derived from the TCGA data portal (http://tcga.cancer.gov), which is a publicly available database.

## Supplementary Materials

Supplementary materials Table S1: forty-three differentially expressed genes significantly related to overall survival in the log-rank test.

## References

[1] Siegel R. L., Miller K. D., Jemal A. (2018). Cancer statistics, 2018. *CA: a Cancer Journal for Clinicians*.

[2] Chow W. H., Dong L. M., Devesa S. S. (2010). Epidemiology and risk factors for kidney cancer. *Nature Reviews. Urology*.

[3] Sejima T., Iwamoto H., Masago T. (2013). Oncological and functional outcomes after radical nephrectomy for renal cell carcinoma: a comprehensive analysis of prognostic factors. *International Journal of Urology*.

[4] Bukowski R. M. (2010). Metastatic clear cell carcinoma of the kidney: therapeutic role of bevacizumab. *Cancer Management and Research*.

[5] Gupta K., Miller J. D., Li J. Z., Russell M. W., Charbonneau C. (2008). Epidemiologic and socioeconomic burden of metastatic renal cell carcinoma (mRCC): a literature review. *Cancer Treatment Reviews*.

[6] Frank I., Blute M. L., Cheville J. C., Lohse C. M., Weaver A. L., Zincke H. (2002). An outcome prediction model for patients with clear cell renal cell carcinoma treated with radical nephrectomy based on tumor stage, size, grade and necrosis: the SSIGN score. *The Journal of Urology*.

[7] Yoshihara K., Shahmoradgoli M., Martínez E. (2013). Inferring tumour purity and stromal and immune cell admixture from expression data. *Nature Communications*.

[8] Mier J. W. (2019). The tumor microenvironment in renal cell cancer. *Current Opinion in Oncology*.

[9] Cooper L. A. D., Gutman D. A., Chisolm C. (2012). The tumor microenvironment strongly impacts master transcriptional regulators and gene expression class of glioblastoma. *The American Journal of Pathology*.

[10] Galon J., Pages F., Marincola F. M. (2012). The immune score as a new possible approach for the classification of cancer. *Journal of Translational Medicine*.

[11] Winslow S., Lindquist K. E., Edsjo A., Larsson C. (2016). The expression pattern of matrix-producing tumor stroma is of prognostic importance in breast cancer. *BMC Cancer*.

[12] Petitprez F., Sun C. M., Lacroix L., Sautes-Fridman C., de Reynies A., Fridman W. H. (2018). Quantitative analyses of the tumor microenvironment composition and orientation in the era of precision medicine. *Frontiers in Oncology*.

[13] Fridman W. H., Pages F., Sautes-Fridman C., Galon J. (2012). The immune contexture in human tumours: impact on clinical outcome. *Nature Reviews. Cancer*.

[14] Ni J., Liu S., Qi F. (2020). Screening TCGA database for prognostic genes in lower grade glioma microenvironment. *Annals of Translational Medicine*.

[15] Meng Z., Ren D., Zhang K., Zhao J., Jin X., Wu H. (2020). Using ESTIMATE algorithm to establish an 8-mRNA signature prognosis prediction system and identify immunocyte infiltration-related genes in pancreatic adenocarcinoma. *Aging (Albany NY)*.

[16] Zhou L., Huang W., Yu H.-F., Feng Y.-J., Teng X. (2020). Exploring TCGA database for identification of potential prognostic genes in stomach adenocarcinoma. *Cancer Cell International*.

[17] Verhaak R. G. W., Hoadley K. A., Purdom E. (2010). Integrated genomic analysis identifies clinically relevant subtypes of glioblastoma characterized by abnormalities in PDGFRA, IDH1, EGFR, and NF1. *Cancer Cell*.

[18] Barbie D. A., Tamayo P., Boehm J. S. (2009). Systematic RNA interference reveals that oncogenic KRAS-driven cancers require TBK1. *Nature*.

[19] Ritchie M. E., Phipson B., Wu D. (2015). Limma powers differential expression analyses for RNA-sequencing and microarray studies. *Nucleic Acids Research*.

[20] Banat G. A., Tretyn A., Pullamsetti S. S. (2015). Immune and inflammatory cell composition of human lung cancer stroma. *PLoS One*.

[21] Jia D., Li S., Li D., Xue H., Yang D., Liu Y. (2018). Mining TCGA database for genes of prognostic value in glioblastoma microenvironment. *Aging (Albany NY)*.

[22] Mao Y., Keller E. T., Garfield D. H., Shen K., Wang J. (2013). Stromal cells in tumor microenvironment and breast cancer. *Cancer Metastasis Reviews*.

[23] Hamada S., Masamune A., Shimosegawa T. (2013). Alteration of pancreatic cancer cell functions by tumor-stromal cell interaction. *Frontiers in Physiology*.

[24] Banerjee S., Modi S., McGinn O. (2016). Impaired synthesis of stromal components in response to Minnelide improves vascular function, drug delivery, and survival in pancreatic cancer. *Clinical Cancer Research*.

[25] Allavena P., Mantovani A. (2012). Immunology in the clinic review series; focus on cancer: tumour-associated macrophages: undisputed stars of the inflammatory tumour microenvironment. *Clinical and Experimental Immunology*.

[26] Bingle L., Brown N. J., Lewis C. E. (2002). The role of tumour-associated macrophages in tumour progression: implications for new anticancer therapies. *The Journal of Pathology*.

[27] Li X., Wenes M., Romero P., Huang S. C.-C., Fendt S.-M., Ho P.-C. (2019). Navigating metabolic pathways to enhance antitumour immunity and immunotherapy. *Nature Reviews. Clinical Oncology*.

[28] Hu D., Zhou M., Zhu X. (2019). Deciphering immune-associated genes to predict survival in clear cell renal cell cancer. *BioMed Research International*.

[29] Dong L. M., Brennan P., Karami S. (2009). An analysis of growth, differentiation and apoptosis genes with risk of renal cancer. *PLoS One*.

[30] Wu J., Zhang Y., Frilot N., Kim J. I., Kim W. J., Daaka Y. (2011). Prostaglandin E2 regulates renal cell carcinoma invasion through the EP4 receptor-Rap GTPase signal transduction pathway. *The Journal of Biological Chemistry*.

[31] Yeung R. S., Xiao G. H., Jin F., Lee W. C., Testa J. R., Knudson A. G. (1994). Predisposition to renal carcinoma in the Eker rat is determined by germ-line mutation of the tuberous sclerosis 2 (TSC2) gene. *Proceedings of the National Academy of Sciences of the United States of America*.

[32] Hong S. B., Oh H., Valera V. A. (2010). Tumor suppressor FLCN inhibits tumorigenesis of a FLCN-null renal cancer cell line and regulates expression of key molecules in TGF-*β* signaling. *Molecular Cancer*.

