# Supplementary material for: Identification of Key Genes of Prognostic Value in Clear Cell Renal Cell Carcinoma Microenvironment and a Risk Score Prognostic Model
Source: Dis Markers. 2020 Sep 3;2020:8852388. doi: 10.1155/2020/8852388 (PMC7487089; doi:10.1155/2020/8852388)
Supplement: Supplementary materials — Table S1: forty-three differentially expressed genes significantly related to overall survival in the log-rank test. [file 8852388.f1.docx]

Table S1 Forty-three differentially expressed genes significantly related to overall survival in log-rank test

| Gene | P value |
| --- | --- |
| PAEP | 6.38E-08 |
| SLC22A6 | 5.23E-07 |
| OGDHL | 1.06E-06 |
| GJB6 | 6.62E-06 |
| SLN | 1.05E-05 |
| OBP2A | 1.24E-05 |
| LDHD | 1.51E-05 |
| ADGRV1 | 2.06E-05 |
| APCDD1L | 5.16E-05 |
| SLC22A8 | 5.71E-05 |
| CPA4 | 8.54E-05 |
| CWH43 | 0.000101 |
| PPARGC1A | 0.000119 |
| HMGCS2 | 0.000128 |
| SLC22A12 | 0.000138 |
| AQP9 | 0.000144 |
| FDCSP | 0.000158 |
| GPAT3 | 0.000165 |
| TNFSF13B | 0.000187 |
| FREM1 | 0.000198 |
| HSD11B2 | 0.000912 |
| MIXL1 | 0.001078 |
| FCRL5 | 0.001128 |
| GREM1 | 0.003341 |
| MZB1 | 0.003759 |
| XCR1 | 0.003805 |
| ZPLD1 | 0.003835 |
| CASP5 | 0.005558 |
| TMEM38A | 0.005956 |
| CHRDL2 | 0.007055 |
| RORB | 0.008096 |
| IGLL5 | 0.009196 |
| PAH | 0.010551 |
| MUC20 | 0.011627 |
| SCARA5 | 0.018495 |
| KCNJ11 | 0.019569 |
| IL10 | 0.027363 |
| HSD11B1 | 0.02799 |
| VSIG4 | 0.029019 |
| F7 | 0.036674 |
| RAP1GAP | 0.039012 |
| POU2AF1 | 0.043038 |
| KLK3 | 0.044419 |
